# Analysis of Turkish Breast Cancer Patients With ATM-Heterozygous Germline Mutation According to Clinicopathological Features

**DOI:** 10.7759/cureus.47324

**Published:** 2023-10-19

**Authors:** Oktay Ünsal, Büşra Güvercin, Ahmet Özet, Mehmet Ali Ergün

**Affiliations:** 1 Department of Medical Oncology, Gazi University Faculty of Medicine, Ankara, TUR; 2 Department of Internal Medicine, Gazi University Faculty of Medicine, Ankara, TUR; 3 Department of Medical Genetics, Gazi University Faculty of Medicine, Ankara, TUR

**Keywords:** turkish population, heterozygous, germline mutation, atm, breast cancer

## Abstract

Objective: The ATM gene is one of the most common breast cancer (BC) susceptibility genes after BRCA1/2 and has been shown to be a moderate BC susceptibility gene. The association between ATM germline mutation and clinical features of BC is now unknown. In this article, clinicopathological features of BC patients with ATM germline heterozygous mutation were investigated.

Materials and methods: Patients admitted to the Medical Genetics department of a tertiary hospital between January 2020 and December 2022 were examined. Only invasive BC patients with pathogenic mutation, likely pathogenic mutation, or variants of uncertain significance (VUS) were included in the study.

Results: In all, 121 patients were included in the study. The median age at the first cancer diagnosis of the patients was 44 years. Of the total number of patients, 75.2% (91) had the histological subtype of infiltrating ductal carcinoma, and 43% (52) had Luminal B molecular subtype features. At a median follow-up of 16 months, 5.8% (7) of patients developed cancer in the contralateral breast. In addition, 7.4% (9) of the patients developed a second primary cancer during follow-up. When the patients were compared according to ATM variant classification, the localization, histologic types, and molecular subtypes of the BC were not different between all groups (respectively; *p*=0.68, *p*=0.65, *p=*0.32).

Conclusions: To the best of our knowledge, this is the first publication that evaluates the clinical and pathological characteristics of BC patients with germline heterozygous ATM mutations in the Turkish population. When patients were compared according to variant classifications of ATM mutation, patients' histological and molecular subtypes were similar.

## Introduction

Compound heterozygous or homozygous mutations in the A-T mutation (ATM) gene are the main causes of ataxia-telangiectasia (A-T). The ATM gene is located on chromosome 11q22-23 and is one of the most common breast cancer (BC) vulnerability genes after BRCA1/2 [1].

ATM gene plays a main role in repairing DNA double-strand breaks, which can occur as a result of oxidative stress, chemotherapy drugs, ionizing radiation, or normal physiological events, such as rearrangement of antibody genes during B-cell maturation [2,3]. After DNA damage occurs, ATM phosphorylates BRCA1, TP53, and numerous proteins included in the DNA double-strand break (DSB) respond. Hereditary mutations of genes involved in DNA repair lead to vulnerability to various malignancies. People who are carriers of mutated ATM genes have a higher risk of vulnerability to BC and other malignant diseases [4,5]. ATM has been shown to be a moderate BC susceptibility gene [6]. While the risk of BC in heterozygous ATM mutation carriers is 6% until the age of 50, this rate increases to 33% until the age of 80 [7].

The association between ATM germline mutation and clinical features is not known. There are few studies in the literature investigating the histopathological features of BC developed by heterogeneous ATM variants. Renault et al. demonstrated that the majority of ATM-associated tumors were luminal B tumors [8]. Furthermore, Yang et al. compared patients with BC with and without ATM germline mutation and it was seen that the rate of progesterone receptor (PR) and estrogen receptor (ER) positivity were higher in patients with pathogenic mutations [9]. In another study, invasive ductal BC was the most common histological subtype for ATM pathogenic variant (PV) carriers [10].

ATM, along with BRCA1, BRCA2, CHEK2, TP53, and several other genes included in BC susceptibility, is nowadays being tested in most gene panel analyses for BC patients. With the use of multi-gene panel testing for BC-associated mutations, new challenges have been revealed in the follow-up of individuals at high risk of cancer and cancer patients. In addition, the lack of clear guidelines for moderate and low penetration genes and the increasing frequency of variants of uncertain significance (VUS) in panel testing poses challenges for counseling and risk management [11,12].

The objective of our study was to examine the clinicopathological characteristics of BC patients with germline heterozygous ATM variants. Also, we aimed to compare some clinical and pathological characteristics of the patients according to ATM variant classification.

## Materials and methods

Study population

Patients admitted to the Department of Medical Genetics of a tertiary hospital between January 2020 and December 2022 were retrospectively examined. Among these patients, patients with PV, likely pathogenic (LP), and VUS in germline ATM heterozygous mutation gene, and patients with invasive BC were examined in the study. Patients younger than 18 years of age, those with a diagnosis of carcinoma in situ, those with somatic mutations, and patients with solid cancer other than BC were excluded. The hospital data of 121 patients who met these criteria were reviewed. A total of 121 patients with PV, LP, and VUS ATM mutations were evaluated. The study protocol was approved by the ethics committee of Gazi University Faculty of Medicine (2023-397).

Pathology

The human epidermal growth factor receptor 2 (HER2), ER, and PR were analyzed using an immunohistochemical (IHC) test on BC tissue acquired from core needle biopsies or surgery resected material. While ER or PR positivity was described as ≥ 1% of tumor cells with positive nuclear staining, HER2 positivity was described as a score of 3+ or by HER-2 gene amplification using fluorescent in situ hybridization.

Tumors were classified as follows: luminal A (ER+, PR+/−, HER2− and Ki-67 < 20%), and luminal B (ER+, PR+/−, HER2− and Ki-67 ≥ 20%), HER2 overexpressing (ER-, PR- and HER2+), triple negative (ER-, PR- and HER2-) [13].

ATM mutation screening

Peripheral blood samples were taken into ethylenediaminetetraacetic acid (EDTA) tubes based on a recent revision of the Declaration of Helsinki. Sequencing libraries were prepared using the CE-IVD SOPHiA HCS v1.1 kit. Samples were sequenced according to the protocols of SOPHiA GENETICS. The SOPHiA HCS allows for the enrichment of coding and splicing regions of 26 genes (ATM, CHEK2, APC, PIK3CA, BRCA1, BRCA2, MUTYH, PALB2, TP53, PTEN, STK11, BARD1, BRIP1, CDH1, MLH1, MSH2, MSH6, EPCAM, FAM175A, MRE11A, NBN, PMS2, RAD50, RAD51C, RAD51D, XRCC2). The sequencing data were simultaneously processed for copy number variations (CNVs), single nucleotide variants (SNVs) and indels using the SOPHiA DDM software updated to the recent version at the time of sequencing.

Mutation classification

Variants have been reported in 5 classes (PV, LP, VUS, Likely Benign, and Benign) in accordance with international standards [14]. PV, LP and VUS variants of these variants were included in the study.

Statistical analysis

The normality test was performed for the presence of normal distribution. Due to non-normal distribution, the age of the patients was noted using medians. The age of the patients was noted using medians. The presence of family history, localization of BC, histologic and molecular types, and ATM variant classification were shown in numbers and percentages. Comparison of some clinical and laboratory parameters of the patients according to ATM variant classification was performed using the Kruskal-Wallis test, the chi-squared test, or Fisher's exact test, where appropriate. A p-value of less than 0.05 was thought to be associated with a statistically significant result.

## Results

All of 121 patients enrolled in the study were female. The median age of the study population was 48 (32-68) years. Of all patients, 75.2% (91) had the histological subtype of infiltrating ductal carcinoma, and 43% (52) had Luminal B molecular subtype features. According to variant classification, it was seen that VUS was the most common variant (57.9%-70 patients). When the localization of BC was evaluated, right or left localizations were seen to be similar. Other baseline characteristics of the patients are presented in Table 1.

**Table 1:** Baseline characteristics of the study population ATM: ataxia telangiectasia mutated, HER2: human epidermal growth factor receptor 2, VUS: variant of uncertain significance *: tubular carcinoma, mucinous (colloid) carcinoma, medullary carcinoma, tubulolobular carcinoma Data presented as n-% except the age (noted using medians)

Parameters		Values
Age (years)		48 (32-68)
Presence of cancer in family history (n-%)		80 (66.1)
Localization (n-%)	Right	61 (50.4)
	Left	60 (49.6)
Histologic types (n-%)	Infiltrating ductal carcinoma	91 (75.2)
	Infiltrating lobular carcinoma	21 (17.4)
	Mixed ductal/lobular carcinoma	2 (1.7)
	Others*	7 (5.7)
Molecular subtypes (n-%)	Luminal A	38 (31.4)
	Luminal B	52 (43)
	HER2-enriched	24 (19.8)
	Basal subtypes	7 (5.8)
ATM variant classification (n-%)	Pathogenic	36 (29.7)
	Likely pathogenic	15 (12.4)
	VUS	70 (57.9)

The median age at first cancer diagnosis was 44 years (31-67). The median follow-up period for BC was 16 (6-247) months. Patients who developed cancer in the contralateral breast at follow-up were 5.8% (7 patients) of all patients (two of them were synchronous). The median time to develop cancer in the contralateral breast was 60 (0-219) months. Second primary cancer was developed in 7.4% (9 patients) of the patients during follow-up. The second primary most common cancer was thyroid papillary carcinoma which developed in 4 patients (3.3%).

While most of the PV was right localized (19 patients, 52.8%), most LP was seen to be localized left (9 patients, 60%) (Table 2). The most common histological subtype was infiltrating ductal carcinoma in all ATM variant groups. Their percentages were as follows; 30 PV (85.7%), 9 LP (75%), and 52 VUS (80%). ). In the PV and LP groups, the patients with the Luminal B subtype were more common (respectively; 19 patients at 52.8%), and 8 patients at 53.3%). Also, in VUS variants, it was seen that the Luminal A and B subtypes were found to be similar percentages (25 patients, 35.7%) (Table 2).

**Table 2:** Comparison of some clinical and laboratory parameters of the patients according to ATM variant classification VUS: variant of uncertain significance *: Due to small numbers, mixed ductal/lobular carcinoma and others—tubular carcinoma, mucinous (colloid) carcinoma, medullary carcinoma, and tubulolobular carcinoma—were not included in the statistical analysis Data presented as n-% and p<0.05 is considered significant

Parameters		ATM variant classification			p value
		Pathogenic (n=36)	Likely pathogenic (n=15)	VUS (n=70)	
Localization (n-%)	Right	19 (52.8)	6 (40)	36 (51.4)	0.68
	Left	17 (47.2)	9 (60)	34 (48.6)	
Histologic types (n-%) *	Infiltrating ductal carcinoma	30 (85.7)	9 (75)	52 (80)	0.65
	Infiltrating lobular carcinoma	5 (14.3)	3 (25)	13 (20)	
Molecular subtypes (n-%)	Luminal A	9 (25)	4 (26.7)	25 (35.7)	0.32
	Luminal B	19 (52.8)	8 (53.3)	25 (35.7)	
	HER2-enriched	6 (16.7)	1 (6.7)	17 (24.3)	
	Basal subtypes	2 (5.6)	2 (13.3)	3 (4.3)	

When the patients were compared according to ATM variant classification, the localization, histologic types, and molecular subtypes of the BC were not different between the three groups (respectively; p=0.68, p=0.65, p=0.32) (Table 2). Although tumor localizations were similar in all ATM variant groups, it was slightly more localized in the left breast in the LP group (Table 2).

## Discussion

In this study, clinicopathological features of a total of 121 patients with germline heterozygous ATM mutation, including PV, LP, and VUS groups, were presented. We demonstrated that infiltrating ductal carcinoma was the most common histological subtype and Luminal B was the most common molecular subtype. When the patients were compared according to ATM variant classifications, we found that the histological and molecular subtypes of the patients were similar. On the other hand, when ATM variants were compared according to tumor localization, left BC was numerically more frequent in the LP group, but it was not statistically significant. At a median 16-month follow-up, 5.8% (7 patients) of patients developed contralateral BC. In addition, we observed that 7.4% (9 patients) of the patients developed second primary cancer during the follow-up period.

The ATM gene is related to proteins that act as tumor suppressors and are included in the DNA damage response after the generation of DNA DSBs [15]. Germline ATM heterozygous is detected in approximately 1% of the population [16]. Studies in the literature on relatives with heterozygous ATM gene mutations have shown an approximately 2-3 times increased risk of cancer in women and a 5-9 times increased risk of BC [16,17]. Thompson et al. demonstrated that the risk of developing BC is higher in heterozygous ATM carriers, especially before the age of 50 [18]. In the current study, the median age at the first diagnosis of BC was 44 years, which is consistent with the literature [19]. On the other hand, in the studies, there are conflicting findings on the role of ATM mutations in increasing the risk of contralateral BC [19,20]. Although evidence is conflicting if the contralateral prophylactic mastectomy improves survival for BRCA carriers with BC, this approach reduces the risk of contralateral tumors by 93% [21]. There is no study investigating risk reduction and survival advantage in relation to contralateral prophylactic mastectomy for patients with BC harboring ATM LP or PV. In our study population, 5.8% (7 patients) of patients developed contralateral BC at a median follow-up of 16 months.

As our knowledge of the genetic heterogeneity of BC has increased, evidence has emerged that mutations in different genes may be associated with different BC subtypes. While BRCA1 mutations are known to be associated with triple-negative BC [22], TP53 mutations are known to be associated with HER2-positive BC [23]. Renault et al. examined BC developing in ATM carriers and they demonstrated that the molecular subtype of tumors, most commonly associated with ATM, was luminal B tumors [8]. In the current study, Luminal B patients were more common in both PV and LP groups. Although ATM-associated tumors are mostly luminal tumors like CHEK2 and BRCA2-associated tumors [24,25], they do not show a particular histological subtype as observed in BRCA1 (medullary), BRCA2 (lobular), PTEN-associated tumors (apocrine), CDH1 (lobular) [26-28]. As with BRCA-associated tumors, identification of the clinical and biological features of ATM-associated tumors can help us to improve diagnosis, prognosis, and targeted therapeutic approaches [29,30].

According to the 2023 National Comprehensive Cancer Network guidelines, patients who are heterozygous for a PV or LP ATM mutation should undergo annual mammographic screening starting at least 40 years of age and contrast-enhanced breast MRI screening starting from 30-35 years of age. Because these individuals have a lifetime risk of BC likely greater than 20%.

The phenotypes of BRCA-associated tumors have been widely described, but insufficient information is available on the clinical and pathological features of ATM-associated BCs. To the best of our knowledge, this is the first publication that examines the clinical and molecular characteristics of BC patients with germline heterozygous ATM mutations in the Turkish population. It should be noted that although the study group consists of patients from different regions of Turkey, it does not represent the entire Turkish population. In societies with high consanguineous marriage rates, such as Turkey, germline genetic testing improves risk assessment. It influences decisions about prophylactic interventions, surveillance, and treatment options for patients and their at-risk relatives. These findings provide healthcare providers and health authorities with valuable data that can be useful for decision-making, genetic counseling, or policy-making in genetic testing for different patient groups.

There were some limitations of our study. Firstly, due to its retrospective design, there was insufficient data. Also, the majority of our study population was patients with VUS variants which may affect our results. Further evaluation with a larger population may demonstrate valuable results.

## Conclusions

This study investigated germline ATM mutations in patients with BC. This is the first publication that evaluates the clinical and pathological characteristics of BC patients with germline heterozygous ATM mutations in the Turkish population. The most common histological subtype was infiltrative ductal carcinoma, and the most common molecular subtype was Luminal B. When patients were compared according to variant classifications of ATM mutation, patients' histological and molecular subtypes were similar.
